# Diagnostic Performance of Theranostic Radionuclides Used in Transarterial Radioembolization for Liver Cancer

**DOI:** 10.3389/fonc.2020.551622

**Published:** 2021-01-25

**Authors:** Rou Li, Danni Li, Guorong Jia, Xiao Li, Gaofeng Sun, Changjing Zuo

**Affiliations:** ^1^ Department of Nuclear Medicine, Shanghai Changhai Hospital, Shanghai, China; ^2^ School of Medical Imaging, Xuzhou Medical University, Xuzhou, China

**Keywords:** liver cancer, diagnostic performance, theranostic radionuclides, transarterial radioembolization, nuclear imaging

## Abstract

Primary liver tumor with hepatocellular carcinoma accounting for 75–80% of all such tumors, is one of the global leading causes of cancer-related death, especially in cirrhotic patients. Liver tumors are highly hypervascularized *via* the hepatic artery, while normal liver tissues are mainly supplied by the portal vein; consequently, intra-arterially delivered treatment, which includes transarterial chemoembolization (TACE) and transarterial radioembolization (TARE), is deemed as a palliative treatment. With the development of nuclear technology and radiochemistry, TARE has become an alternative for patients with hepatic cancer, especially for patients who failed other therapies, or for patients who need tumor downstaging treatment. In practice, some radionuclides have suitable physicochemical characteristics to act as radioactive embolism agents. Among them, ^90^Y emits β rays only and is suitable for bremsstrahlung single photon emission computed tomography (BS SPECT) and positron emission tomography (PET); meanwhile, some others, such as ^131^I, ^153^Sm, ^166^Ho, ^177^Lu, ^186^Re, and ^188^Re, emit both β and γ rays, enabling embolism beads to play a role in both therapy and single photon emission computed tomography (SPECT) imaging. During TARE, concomitant imaging provide additive diagnostic information and help to guide the course of liver cancer treatment. Therefore, we review the theranostic radionuclides that have been used or could potentially be used in TARE for liver cancer and focus on the clinical benefits of diagnostic applications, including real-time monitoring of embolism beads, evaluating irradiation dose, predicting therapy effects, and corresponding adjustments to TARE.

## Introduction

The primary liver tumor often occurs in patients with liver cirrhosis, with hepatitis virus being the most common risk factor for liver cirrhosis (1). As the most common type of liver cancer, hepatocellular carcinoma (HCC) is the fifth most common cancer worldwide and the second leading cause of cancer-related mortality (2).

Nowadays, there are many options for the treatment of liver cancer according to the guidelines in the Barcelona Clinic Liver Cancer (BCLC) algorithm (3). However, because of the underlying liver dysfunction and its often-late diagnosis, only approximately 30% of patients when initially diagnosed are eligible for curative treatments (resection, percutaneous ablation, etc). Additionally, due to locally advanced diseases, poor liver function, or additional comorbidities, recurrence or metastasis are common after major curative treatment. Hence, various palliative treatments are considered for intermediate-advanced stage liver cancer, especially in inoperable cases. Taking advantage of the fact that the tumor is supplied by the hepatic artery while the surrounding normal liver tissue is mainly supplied by the portal system, transarterial radioembolization (TARE) appears as one of the attractive treatment options.

TARE intra-arterially delivers therapeutic radioactive agents to the tumor, which can achieve embolization of the feeding artery and radiation killing effect on tumor cells simultaneously during the treatment of liver cancer. Different from external radiotherapy, TARE limits systemic irradiation and preserves the healthy liver to a maximum extent. Additionally, the incidence of postembolization syndrome in TARE is relatively rare and mild compared to TACE (transarterial chemoembolization). TARE has been demonstrated to be feasible in treating patients at different stages or with postoperative recurrence, and serves as a downstaging strategy prior or as a bridge to transplantation (4). Meanwhile, TARE is an alternative treatment for those may be contraindication to other treatments.

Prior to TARE, structural imaging, such as computed tomography (CT) or magnetic resonance (MR), shows the size and location of the tumor and its relationship with the surrounding tissue. Besides, x-ray angiography is also vital before TARE, which can provide the blood supply of the tumor and the travel, distribution and variation of blood vessels. Single photon emission computed tomography/computed tomography (SPECT/CT) is also common imaging method during TARE. All these imaging technologies are helpful to guide treatment planning (e.g., the method of delivery, suitable treatment dose) and even predict treatment efficacy. After injecting the theranostic radionuclide into the hepatic artery, therapeutic effect is achieved by internal exposure around the tumor. Meanwhile, real-time or post-therapeutic nuclear imaging, such as SPECT/CT, provides information on verification of treatment delivery, the biodistribution of radioconjugates, integral radiation dose at tumor site and organs of interest (lung, liver, thyroid gland, etc) and prediction of treatment outcome. Later post-therapeutic imaging mainly observes the therapeutic effect on the tumor and monitors other organs for complications. Furthermore, positron emission tomography/computed tomography (PET/CT) is also a potential imaging modality for TARE. It realizes functional imaging before and after treatment by showing the glucose metabolism of the tumor and sensitively finding metastasis. Hence, imaging plays a key role in drug development and clinical evaluation, and is conducive to developing adaptive TARE plans according to the metabolic mode.

Theranostic radionuclides integrating imaging and treatment during radioembolization are the most important component of TARE for liver cancer. Based on how TARE works, radionuclides that emit α rays are not suitable because of their pathlengths are relatively short and thus have a limited range in tissue. Radionuclides emiting β rays can provide substantial radiation therapy for liver tumors due to their pathlengths ranging from millimeters to centimeters. Meanwhile, emitted γ rays are helpful in monitoring embolization beads using nuclear imaging. Considering the therapeutic purpose, the half-life of nuclides ranging from one to several days is suitable, allowing the tumor to be exposed to sufficient radiation while avoiding radiation damage in the process of excretion. Thus far, only two ^90^Y based microspheres (MSs) and ^131^I-lipiodol are approved for clinical use, while many others are still in the pre-clinical or basic research stage. Recently, the use of additional theranostic nuclides in TARE has been explored. **Table 1** summarizes the critical characteristics (nuclear property and imaging type) of theranostic nuclides that have been used and those that have shown potential in TARE for hepatic cancer. At the same time, the theranostic effects, especially the diagnostic performance of those theranostic radionuclides will be summarized by nuclides and presented here. Among them, ^90^Y is the most important.

**Table 1:** Major radionuclides used for radioembolization of hepatocellular carcinoma.

Radionuclides	Emission and energy (MeV)	Half-life	Maximum tissue penetration (mm)	Imaging type
^90^Y	β 2.28 (100%) γ/	2.7 d	12	BS SPECT/ PET
^32^P	β 1.71 (100%) γ/	14.3 d	7.9	BS SPECT/ PET
^131^I	β 0.607 (99%) γ 0.364 (1%)	8.04 d	2	SPECT
^188^Re	β 2.12 (71.6%) 1.96 (25.1%) γ 0.155 (15%)	16.9 h	11	SPECT
^166^Ho	β 1.84 (50.5%) 1.74 (48.7%) γ 0.081 (6.2%) 0.00138(0.93%)	1.1 d	8.7	SPECT/MR
^177^Lu	β 0.176 (12.2%) 0.497 (78.6%) 0.384 (9.1%) γ 0.113 (6.4%) 0.208 (11%)	6.7 d	2.2	SPECT
^153^Sm	β 0.81 (20%) 0.71 (30%) 0.64 (50%) γ 0.103 (28%)	46.3 h	3	SPECT
^186^Re	β 2.13 (70%) 1.98 (26%) γ 0.155 (16.2%) 0.633 (1.6%)	3.8 d	4.5	SPECT

## Yttrium-90

Yttrium-90, which has favorable physicochemical characteristics, is presently the most widely used nuclide for TARE of liver cancer clinically. Yttrium-90 is a pure β emitter and has a half-life of 64.2 h. The average energy of β emission is 0.937 MeV with a mean tissue penetration of 2.5 mm. High energy, short tissue penetration distance and relatively short half-life make ^90^Y a suitable nuclide in TARE. Compared with other radionuclide compounds, ^90^Y-labeled MSs (^90^Y-MSs) are already clinically available for liver cancer in some countries. The two currently commercially available ^90^Y-MS products are glass MSs (TheraSphere) and resin MSs (SIR-Spheres). Because of the different physical and chemical properties, the treatment dose calculation for ^90^Y-MS TARE differs significantly according to the MS type (5).

Blood supply and hepatic collateral circulation of tumor are essential for the efficacy and safety of treatment. Current clinical ^90^Y-MS BS SPECT (bremsstrahlung single photon emission computed tomography) is hindered by image quality, while ^90^Y-MS PET is extremely challenging due to low activity concentrations in the lungs and the low positron yield of ^90^Y. Hence, ^99m^Tc-MAA (Technetium-99m macroaggregated albumin) is a surrogate of ^90^Y for pre-treatment imaging. Intra-arterially delivery of ^99m^Tc-MAA is able to simulate the distribution of therapeutic drugs in the liver, by measuring the radiation intensity of cancerous and non-cancerous liver tissue respectively, the ratio of tumor/non-tumor (T/NT) can be calculated more accurately (6). That provides a quantitative index for evaluating the abundance of tumor blood supply and the ability of cancer tissue to absorb therapeutic drugs through hepatic artery. Patients should undergo ^99m^Tc-MAA examination before ^90^Y-MS TARE, which offers important preprocedural information. In ^99m^Tc-MAA SPECT/CT, ^99m^Tc-MAA (4–5 mCi) is injected into the hepatic artery to assess extra-hepatic depositions and lung shunting, and can provide the calculation of the lung shunt absorbed dose, which guides dosimetry to avoid radiation pneumonitis. Moreover, accurate superselective angiography should be performed to evaluate the targeted vascular territory and anatomic variants or extra-hepatic vessels should accept prophylactic embolization, which can avoid ectopic embolization of non-targeted organs. However, the concordance between ^99m^Tc-MAA and ^90^Y-MSs remains controversial. Many studies have reported both similarity (7) and differences (8) between ^99m^Tc-MAA and ^90^Y-MSs uptake. The inconsistent concordance may be due to several factors (the different size, shape, and number of MAA relative to microspheres, flow dynamics during delivery, etc), for which the post-therapy images of ^90^Y-activity distribution is considered appropriate to determine the tumor dose-response. Apart from SPECT/CT, CT or MR imaging are also be used to assess the volume, location, and relationship with surrounding hepatic tissues of the tumor before TARE.

^99m^Tc-MAA scanning and superselective angiography are often performed before ^90^Y-MS TARE to guide dosimetry, although accumulating evidence has improved the safety and efficacy of using radioembolization with ^90^Y-labeled resin or glass MSs for the management of hepatic tumors (9). A study of 1,652 patients (1,124 TACE, 528 ^90^Y-TARE) showed that ^90^Y-MS TARE increased the 2-year overall survival (OS) rates relative to the observational subgroup and resulted in better objective response (OR) rates than treatment with TACE. Furthermore, a lower risk of adverse events was observed in ^90^Y-TARE than in TACE (10). Many studies have demonstrated that ^90^Y-MS TARE is an effective choice and results in significantly fewer severe adverse events when treating HCC patients with portal vein thrombosis (PVT), which is a contraindication for most traditional therapies (11).

For post-treatment imaging, compared with the majority of the radionuclides used in TARE, ^90^Y is a pure β emitter and lacks discrete-energy photon emissions, such as γ rays and/or characteristic fluorescence x-rays (12). The β particles emitted by ^90^Y produce secondary bremsstrahlung radiation, which may be imaged to locate the ^90^Y-MSs. Commonly, BS SPECT is used after radioembolization to exclude extra-hepatic activity deposition and to assess intra-hepatic MSs distribution. Ahmadzadehfar et al. performed BS SPECT/CT imaging on 188 patients who accepted ^90^Y-MS TARE 24 h prior, to assess the capability of BS SPECT/CT in detecting and positioning of extrahepatic accumulation (gastrointestinal complications). Gastroduodenoscopy served as reference standards. The results showed that the positive predictive values, sensitivity, specificity and the accuracy of BS SPECT/CT were 100, 87, 100, and 99%, respectively (13). Furthermore, BS SPECT/CT after radioembolization is beneficial to evaluate tumor dose-response characteristics and predict treatment response for the management of patients (14). Potrebko et al. demonstrated that voxel-based dosimetry for ^90^Y microsphere therapy allows for quantitative quality assurance of the delivered treatment using deformable image registration, calculated isodose distributions, and dose-volume histograms (DVHs). Since β‐radiation emitted by ^90^Y interacts with body tissues resulting in bremsstrahlung radiation, SPECT/CT has traditionally been regarded as the gold‐standard modality to image the biodistribution of this radionuclide. However, the limitations of the BS SPECT image are poor spatial resolution (11–15 mm) and limited quantitative accuracy because of the low photon yield and continuous nature of the BS X-ray spectrum. The absence of some correction techniques leads to a further reduction in the image contrast. Many efforts have been made to improve BS SPECT/CT imaging qualitatively and quantitatively, in which Monte Carlo simulation is frequently accepted. However, it is not commercially available and cannot be easily implemented clinically (15).

**Figure 1:**
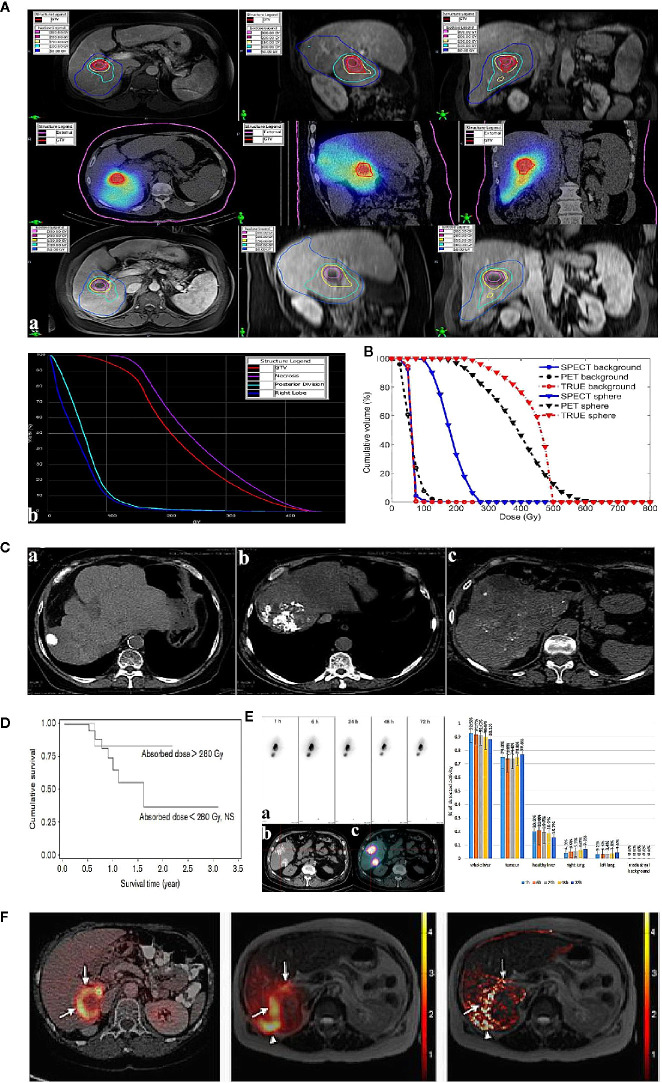
Examples of post-therapeutic imaging of liver tumor. **(A)** The dose of each ROI (regions of interest) in pre-treatment diagnostic MR (top), post-treatment SPECT/CT (middle), and 5-week follow-up MR (bottom) for one patient with HCC (a). Dose-volume histograms of the GTV (gross target volume), necrotic region, posterior division, and right lobe (on the right) (b). Adapted from Potrebko et al. (14). **(B)** CDVH of the phantom (a method of image reconstruction) background ROI and the ROI of the 37-mm diameter sphere. Adapted from Elschot et al. (18). **(C)** Lipiodol tumor accretion at 1 month after intra-arterial injection: complete accretion (49 patients): (a) complete accumulation in 5 patients (10%), (b) partial defect in 15 (31%), (c) faint accumulation in 29 patients (59%). Adapted from Gaultier et al. (19). **(D)** Survival in terms of tumoural absorbed dose (threshold=280 Gy) calculated in tomographic mode. Adapted from Becker et al. (20). **(E)** Intra-arterial injection of ^188^Re-SSS Lipiodol on hepatocellular carcinoma patients. Adapted from Delaunay et al. (21). The left shows whole-body scintigraphic studies (mean geometric) of patient by (a) SPECT, (b) CT, (c) fused SPECT/CT. The right is the average biodistribution profile of patients treated ^188^Re-SSS lipiodol by SPECT/CT image. **(F)** The picture shows intrahepatic biodistribution of ^166^Ho-microspheres on liver tumor patient by ^18^F-FDG PET/CT, SPECT and MR imaging (from left to right). Adapted from Smits et al. (22).

Interestingly, the ^90^Y decay process is of a very small branching to the excited state of stable ^90^Zr. The internal pair production (positron) generated during the decay scheme of ^90^Y allows quantitative monitoring on itself *via* PET (16, 17). Compared with BS SPECT, ^90^Y PET imaging provides better image quality in contrast and resolution (4–5 mm). Elschot et al. quantitatively demonstrated that the image quality of PET was superior to that of BS SPECT for the assessment of ^90^Y-MS distribution after radioembolization in a study of estimated liver dose distributions in 5 patients (18) (**Figure 1B**). The **Figure 1A** showed that the PET-based CDVH (cumulative dose-volume histograms) of the spherical region-of-interest followed the true CDVH more closely than the SPECT-based CDVH. Moreover, calculating the tumor dose on imaging may predict the response to the treatment. A study published in 2013 demonstrated that tumor and non-target tissue absorbed dose quantification using ^90^Y PET was accurate and yielded radiobiologically meaningful dose-response information to guide adjuvant or mitigative action (23). The true definitions of the minimal effective tumor dose and maximum tolerated non-tumor dose remain challenges for each tumor and MS type, and ^90^Y PET may be a potential solution.

Undoubtedly, ^90^Y is the most suitable theranostic radionuclide for TARE of liver cancer so far. ^90^Y, as a pure β emitter, has high energy and relatively small radiation range, which can effectively kill tumor cells while avoiding damage to surrounding healthy tissue, and no requiring subsequent isolation of patients is needed. Although BS SPECT and PET can match the imaging requirements, there are still many unsolved technological issues restricting the accuracy of images and widespread clinical use. For example, low true-coincidence count-rate for positron emission results in relatively longer acquisition times for ^90^Y PET imaging. Besides, standardization and uniform implementation of reconstruction and correction techniques are urgently needed. The necessity of specialized nuclear reactors and relatively long transportation times to each hospital limit the use of ^90^Y. Importantly, ^90^Y is still not be approved and available in many countries, such as in China. Serious complications caused by ectopic embolization of ^90^Y-MS may also occur, which needs high attention in clinical application.

## Iodine-131

The nuclide ^131^I (T_1/2 =_ 8.04d) emits both β and γ rays. Its β particles have a maximum energy of 0.6 MeV and a maximum path length in tissue of 2.3 mm. Utilizing the characteristics of iodine lipiodol, which selectively deposits in nodules of hepatocellular carcinoma, radioactive lipiodol accumulates in cancer tissues and decreases irradiation damage to non-cancer tissues. Hence, ^131^I-lipiodol was often studied for TARE of liver cancer before. In a representative clinical study of 50 patients, the median survival of patients treated with ^131^I-lipiodol was 24 weeks longer than that of the untreated group and no radiotoxic effect was found, especially interstitial pneumonia (19) (**Figure 1C**).

In addition to lipiodol, ^131^I can also be used to label embolism beads, normally in the form of MSs, which are often prepared in stepwise procedures for multiple theranostic applications. Liu et al. incorporated hollow CuS nanoparticles into PLGA (poly lactic-co-glycolic acid) MSs to produce HCuSNPs-loaded MSs, which were then labeled with ^131^I to form ^131^I-HCuSNPs-MSs (24). After the intra-arterial embolization with ^131^I-HCuSNPs-MSs delivered into the hepatic artery of liver tumor-bearing rats, SPECT imaging confirmed that MSs were mainly located in liver tumor and only minimal accumulation was detected in other organs, particularly in the lungs. SPECT imaging findings qualitatively manifested the success of TARE operation and avoidance of damage potentially resulting from the leakage of embolism beads. The follow-up SPECT scan proved that effective embolization lasted for up to 48 h. The predictive results aided understanding of the entire treatment process and prognosis, which was more timely to figure out the primary outcome than traditional follow-up diagnosis, such as blood tests and CT.

Regarding diagnostic performance, ^131^I-based imaging provides qualitative information on whole-body distribution, including tumor accumulation, and even permits semi-quantitative analysis (25). Becker et al. reported that 7 days after the injection, 61% of ^131^I-lipiodol was distributed in the liver tumor, 23% was distributed in non-tumoral liver, and 16% was distributed in the lungs, which highlighted that the clinical toxicities of ^131^I-lipiodol were more commonly affected the lungs (20). More importantly, tumor radioactivity dosimetry helps to define an absorbed dose threshold, especially when the accumulation of the radioactive embolism agent was recorded dynamically (**Figure 1D**). That would predict a therapeutic effect, thus providing guidance for the subsequent therapeutic approach and optimizing the activity to be injected.

There are still some deficiencies in the TARE transformation of ^131^I. Compared to ^90^Y, possible extra managements are needed for the patient’s excreta (such as urine) due to the longer half-life of ^131^I. Besides, non-therapeutic rays (γ rays) with high energy and long path length probably damage the surrounding normal tissue. In addition, too many scattered rays produced by ^131^I lead to relatively low contrast and resolution of SPECT image that are not conducive to quantitative analysis and barely suitable for qualitative analysis. Moreover, defects in terms of *in vivo* stability still exist in ^131^I compounds produced using iodized oil replacement or labeling methods.

## Other Potential Theranostic Radionuclides for TARE

### Rhenium-188

^188^Re is obtained from ^188^W/^188^Re generators in a convenient and comparatively cheap way, and its radioactivity yield can recover to 80% at 40 h postelution. The product of ^188^Re is ^188^Os by β decay, with a half-life of 16.9 h. ^188^Re with a shorter half-life and similar energy of β rays compared ^90^Y, is a promising radionuclide proposed for TARE of hepatic tumors. Similar to ^131^I, the γ ray emission of ^188^Re allows post-therapeutic SPECT imaging.

Early available ^188^Re embolism agents were the lipiodol conjugates as well. ^188^Re-chelator mixed with lipiodol, such as ^188^Re-HDD/lipiodol, ^188^Re-DEDC/lipiodol, and ^188^Re-SSS/lipiodol, has been tested in humans to date. Some studies showed that TARE with ^188^Re-HDD/lipiodol is a safe, effective, and promising method. Meanwhile, ^188^Re was also used to label MSs as embolism beads. A study of 10 patients (3 patients with unresectable colorectal liver metastases and 7 patients with hepatocellular cancer), who received treatment with ^188^Re-human serum albumin MSs, displayed considerable survival rates and tolerable toxicity (26). Other reported ^188^Re-MSs include ^188^Re-PLGA (27), ^188^Re-poly(L-lactic acid) (PLLA) (28), ^188^Re-glass-MSs, and ^188^Re-resin-MSs.

Regarding diagnostic performance, embolization evaluation and related dosimetry in nuclear imaging are helpful for the treatment plan. In a study of 6 patients with unresectable HCC who accepted intra-arterial ^188^Re-SSS/lipiodol, whole-body planar scintigraphy and SPECT were used during therapy (21) and revealed curative hepatic uptake; 90.7 ± 1.6% of radioactivity was detected in the liver with 74.9 ± 1.8% of detected radioactivity in the tumor and 9.3 ± 1.6% of radioactivity was detected in the lungs (**Figure 1E**). The average T/NT uptake ratio was 42.7 ± 7.8 on SPECT. These quantitative data provided a definite diagnosis to conclude that ^188^Re-SSS/lipiodol had satisfactory embolization characteristics, guaranteeing *in vivo* therapeutic application.

Because of its short half-life, much more activities are needed for ^188^Re than ^90^Y to achieve a comparable therapeutic effect, which may produce acute radiation damage to normal tissue. Notably, ^188^Re labeling relies on a relatively complicated reaction, which partially restricts the synthesis of the ^188^Re embolism agents and clinical transformation.

### Holmium-166

^166^Ho emits high-energy β rays, with a comparatively short half-life (1.1 days) (29). Due to its short half-life, ^166^Ho is suitable for patients in urgent need of downstaging treatment compared to the longer radiation therapy time of ^90^Y. ^166^Ho can be produced by two methods, in a nuclear reactor or by neutron activation of ^164^Dy; hence its production is both fast and relatively cheap. Additionally, its outstanding advantage over ^90^Y, ^131^I, and ^188^Re is that ^166^Ho is paramagnetic and emits low-energy γ radiation, which enables the dual MR and SPECT imaging.

^166^Ho-labeled lipiodol has rarely been reported. Das et al. prepared radiolabeled lipiodol by dispersing ^166^Ho-oxine complex in lipiodol (30). For *in vivo* evaluation, the imaging studies of ^166^Ho-lipiodol revealed satisfactory hepatic retention and insignificant uptake was detected in other major organs or tissues except skeleton. ^166^Ho-MSs were extensively studied. Several ^166^Ho-MSs have been prepared so far, including glass, resin, phosphate, and polymer MSs, and all of them have shown good prospects for TARE, especially polymer MSs, due to the advantage of near-plasma density, biodegradability, and biocompatibility (31). In a phase I trial including 15 patients with an unresectable and chemorefractory liver tumor, ^166^Ho-PLLA-MS TARE was implemented in four cohorts of three to six patients, injecting 20 Gy, 40 Gy, 60 Gy, and 80 Gy, respectively. At 6 weeks, treatment response was one patient with partial response, seven with stable disease and seven with disease progression (32). Similarly, ^166^Ho-chitosan MSs and ^166^Ho-alginate MSs (33) that were prepared for radioembolization behaved feasibly and safely.

What is noteworthy is that, due to favorable nuclear imaging characteristics of ^166^Ho, a scout dose (250 MBq) of ^166^Ho is used as an alternative before ^166^Ho-MS TARE, which is superior in calculating the lung shunt fraction compared to ^99m^Tc MAA (34). Considering controversy over the concordance of ^99m^Tc MAA and ^90^Y-MSs, ^166^Ho itself can be used in pre-treatment imaging, for which pre-treatment imaging planning dosimetry could potentially be more accurate. Regarding diagnostic performance, taking advantage of both SPECT and MR imaging, the estimated radiation absorbed dose could be evaluated more precisely in ^166^Ho-complex liver radioembolization. In 2013, Smits et al. performed a phase I clinical trial to investigate the feasibility of quantitative imaging of ^166^Ho radioembolization in 15 patients with unresectable liver cancer based on SPECT and MR imaging (22). The gross comparison yielded a strong correlation between MR and SPECT imaging and moderate agreement between the absorbed dose in each segment as evaluated based on MR and SPECT imaging (**Figure 1F**). The median overall T/NT ratio was 1.4 based on SPECT (range, 0.9–2.8) and 1.4 based on MR imaging (range, 1.1–3.1). In 40% patients (6/15), all T/NT ratios ≥1, indicating that dosimetry based on ^166^Ho-MS SPECT and MR correlated well for the dose in liver segments and tumor tissue. There is no doubt that the use of two imaging modalities allows more informed decision making regarding treatment.

Due to the shorter half-life and lower energy of β rays of ^166^Ho compared to ^90^Y, similar to ^188^Re, a larger administered doses are needed to achieve the dosimetry equivalent to that of ^90^Y for treatment, which potentially cause acute damage to the surrounding normal tissue.

### Lutecium-177

^177^Lu, a popular theranostic nuclide in clinical studies and recently applied in many contexts, may also be the promising radionuclide in TARE for liver cancer treatment. ^177^Lu has a similar half-life to ^90^Y but emits relatively low energy β rays, which limits the radiation exposure to surrounding tissues. Regarding diagnostic performance, emitted γ rays are convenient for positioning observation and radiation dosimetry. As a potential theranostic radionuclide, ^177^Lu has shown to be advantageous in peptide receptor radionuclide therapy (PRRT) of neuroendocrine tumors and peptide radioligand therapy (PRLT) of prostate cancers (35). As a source for TARE, ^177^Lu still has many issues that need to be addressed in future studies. ^177^Lu is produced using reactor irradiation, but few sources of supply and relatively high cost limit its broad application now.

### Samarium-153

^153^Sm emits both β and γ rays, meeting the requirements for theranostic radionuclides. Recent studies have shown that the absorbed doses of ^153^Sm-labeled MSs in all organs can be controlled below 1 Gy and are safe for surrounding healthy tissues (36). Due to limited reports, its therapeutic potential in cancer treatment has not been widely studied, and its diagnostic performance is still not clear.

### Rhenium-186

^186^Re has an appropriate half-life and emits both β and γ rays. At present, ^186^Re is clinically used to treat bone metastases caused by prostate cancer, rarely used in TARE for liver cancer. Due to its shared chemical properties and referential experience in embolism agent development with ^188^Re, ^186^Re is likely to be an ideal nuclide for TARE.

## Conclusion

The incidence of liver cancer is increasing worldwide; nevertheless, effective therapeutic options are limited, and recurrence is common after preferred suitable treatment. Although TARE is not included in the BCLC staging system guidelines, current ongoing randomized clinical trials suggest that TARE is a safe, feasible, and palliative treatment for liver cancer, especially when other conventional treatments have failed. In some cases, where there may be contraindications for other treatments, TARE will be a safe and effective choice. It can achieve a similar or higher overall survival rate and slow down the progression of the disease compared to other routine treatments in the reported literature. Theranostic radionuclides in TARE allow for controlled high radiation doses to be supplied to hepatic tumors while the adjacent liver is relatively spared. In the meantime, diagnostic performance is attractive in the course of treatment. The qualitative or quantitative detection of effective embolization promotes the development of radioactive embolic agents, and assessment of the accumulated dose can guide the course of treatment using post-treatment partial or full body scan. In fact, due to the differences in the supply of nuclides around the world, the current status of clinical application of each radionuclide is not the same. In future, technical improvements and clinical studies are needed to promote the use of radionuclides in TARE. In terms of technology, the technique of radionuclide labeling, efficiency and technology of the acquisition of β and γ rays, and technology of image reconstruction all could be improved and developed for better diagnostic and therapeutic effect. In addition, besides ^90^Y, other potential nuclides (^166^Ho, ^177^Lu, etc) still lack adequate clinical studies and applications for TARE.

Furthermore, except for theranostic radionuclides, embolism agents are also being widely developed and studied. Various new drug-eluting beads (such as, HepaSpheres, CalliSpheres, TANDEM, etc) have obtained certain verification in clinical trials. Natural biological materials, silk fibroin, is proposed to become a potential embolism agent for the treatment of liver cancer and still in basic research stage. In a word, future efforts should be aimed at personalizing TARE therapy depending on the characteristics of different theranostic radionuclides and embolism agents, which making TARE a more effective and safe treatment for liver cancer.

## Author Contributions

RL wrote the manuscript. RL, DL and GJ were responsible for searching related literature. XL, GS, and CZ were involved in revisions and proof-reading. All authors contributed to the article and approved the submitted version.

## Funding

This work was funded by the National Natural Science Foundation of China (81471714 and 81871390) and National Natural Science Foundation Youth Project (81701761).

## Conflict of Interest

The authors declare that the research was conducted in the absence of any commercial or financial relationships that could be construed as a potential conflict of interest.
